# circFNDC3B promotes esophageal squamous cell carcinoma progression by targeting MYO5A via miR-370-3p/miR-136-5p

**DOI:** 10.1186/s12885-023-11314-2

**Published:** 2023-09-04

**Authors:** Dan Song, Ziqi Ye, Fangyu Chen, Liangliang Zhan, Xinchen Sun

**Affiliations:** 1 Department of Radiation Oncology, Jiangsu Cancer Hospital & Jiangsu Institute of Cancer Research & The Affiliated Cancer Hospital of Nanjing Medical University, No.42, Baiziting, Nanjing, 210009 Jinagsu Province China; 2 Department of Radiation Oncology, The First Affiliated Hospital of Nanjing Medical University, No.300, Guangzhou Road, Nanjing, 210029 Jiangsu China

**Keywords:** circRNA, Actinomycin D, RNase R, Xenograft model, Esophageal squamous cell carcinoma

## Abstract

**Background:**

Esophageal squamous cell carcinoma (ESCC) is a prevalent malignant tumor worldwide. Circular RNA (circRNA) is of great value in tumorigenesis progression. However, the mechanism of circFNDC3B in ESCC remains to be clarified.

**Methods:**

Firstly, the circular characteristics of circFNDC3B were evaluated by Actinomycin D and RNase R measurements. The functions of circFNDC3B in ESCC cells were examined by CCK-8, EdU and flow cytometry. Subsequently, the molecular mechanism of circFNDC3B was explained using luciferase reporter gene detection. Finally, we constructed xenograft model to prove the role of circFNDC3B in vivo.

**Results:**

Our study revealed that circFNDC3B was more stable than its linear RNA and prominently upregulated in ESCC. Functional findings suggested that silencing of circFNDC3B reduced the proliferation and enhanced apoptosis of ESCC cells in vitro. Meanwhile, knockdown of circFNDC3B attenuated tumor progression in vivo. Next, miR-370-3p/miR-136-5p was discovered to bind circFNDC3B. miR-370-3p/miR-136-5p reversed the promotive effect on cell proliferation and the inhibitory effect on cell apoptosis of circFNDC3B. MYO5A was a downstream target of miR-370-3p/miR-136-5p. CircFNDC3B served as a sponge for miR-370-3p/miR-136-5p and alleviated the prohibitory effect of miR-370-3p/miR-136-5p on MYO5A, which accelerated ESCC progression.

**Conclusion:**

circFNDC3B positively adjusted the MYO5A expression via spongy miR-370-3p/miR-136-5p, hence achieving the cancer-promoting effect on ESCC. circFNDC3B was a prospective diagnosis marker for ESCC.

**Supplementary Information:**

The online version contains supplementary material available at 10.1186/s12885-023-11314-2.

## Introduction

Esophageal cancer (EC) is a prevalent malignant tumor worldwide with high incidence and death rate [1]. Esophageal squamous cell carcinoma (ESCC) is one of the most predominant subtypes of EC, accounting for around 80% of cases [2]. The causative factors of ESCC include smoking, drinking alcohol and eating overheated food. The early symptoms of ESCC are not easy to detect and there is also a lack of effective early diagnostic markers. Many patients are in an advanced stage when the disease is discovered, delaying the treatment of the disease [3]. Strategies such as radiotherapy and immunotherapy provide additional options for patient care [4]. Despite the great progress has been made in ESCC therapy with scientific advances, the high incidence and low survival rate of ESCC is still an issue that needs to be addressed urgently [5, 6]. The molecular mechanisms underlying ESCC progression remain to be clarified. Consequently, it is valuable to study novel molecular markers and develop potential targeted therapeutic strategies.

Circular RNA (circRNA) is a kind of endogenous non-coding RNA that exists stably in eukaryotes and is mainly enriched in the nucleus [7]. It is characterized by high stability, tissue specificity and functional diversity. Some studies suggested that circRNAs function across RNA, protein, or as regulators of transcription or splicing [8]. With the advancement of RNA sequencing technology and biological research, an increasing number of circRNAs are being discovered and their roles in diseases are becoming known. For instance, circRNA CDR1as interacted with miR-1270, which enhanced the progression of hepatocellular carcinoma [9]. CircRNA_0000285 was aberrantly highly expressed in cervical cancer tissues, induced proliferation and migration of cervical cancer cells through regulation of FUS [10]. Recently, specific functions of circRNAs were revealed in ESCC. The high expression of circGSK3β was linked to the negative prognosis of ESCC patients [11]. One research reflected that FNDC3B was upregulated in breast cancer tissues and connected with cancer cell migration [12]. circFNDC3B derived from FNDC3B was thought to be involved in cardiac repair [13]. In addition, further studies evidenced that circFNDC3B is involved in carcinogenesis. For example, circFNDC3B expression was significantly reduced in bladder cancer tissues, which was related to poor prognosis [14]. Furthermore, the outcomes of Zeng et al. [15] discovered that circFNDC3B was largely expressed in colorectal cancer tissues and circFNDC3B prevented the malignant progression of colorectal cancer via miR-937-5p/TIMP3. However, the specific mechanism of circFNDC3B in ESCC was not clear.

In summary, the purpose of our research was to analyze the role and specific mechanisms of circFNDC3B in ESCC. Our research revealed that circFNDC3B was significantly upregulated in ESCC and circFNDC3B promoted ESCC progression in vivo and in vitro. Additionally, we clarified that circFNDC3B had a binding site with miR-370-3p/miR-136-5p. Our results declared that circFNDC3B had oncogenic potential and might be a diagnostic marker for ESCC.

## Materials and methods

### Clinical research objects

110 ESCC tissues and matched esophageal paracancerous tissues newly diagnosed as ESCC by pathologists at the First Affiliated Hospital of Nanjing Medical University from 2019 to 2020 were collected. All patients signed informed consents. After surgery, fresh cancer tissue and matched paracancerous tissue were preserved at -80 °C immediately. Meanwhile, the clinical characteristics of these 110 participants were gathered. The research was approved by Ethics Committee of the First Affiliated Hospital of Nanjing Medical University.

### Cells culture and transfection

ESCC cells (OE19, TE-1) and the corresponding regular cells HEEC were purchased from ATCC (Manassas, USA). The above cell culture procedures and medium preparation were carried out according to ATCC website.

Specific circFNDC3B or MYO5A siRNAs were established and produced by GenePharma (Shanghai, China). Recombinant overexpression vectors were constructed by inserting circFNDC3B or MYO5A sequences into pLCDH-ciR, respectively. The si-circFNDC3B or si-MYO5A, pLCDH-circFNDC3B or pLCDH-MYO5A, miR-370-3p/miR-136-5p mimics or miR-370-3p/miR-136-5p inhibitors were transfected into ESCC cells using Lipofectamine 3000 (Thermo Fisher Scientific, USA).

The sh-circFNDC3B lentiviral vector and the corresponding negative control were got from the company (Thermo Fisher Scientific, USA). TE-1 cells in logarithmic growth phase were uniformly inoculated into 6-well plates. Cells were infected according to the lentiviral plasmid operating instructions when cell growth density reached 70–80%. Cells were continuously screened with puromycin for 2 weeks after 48 h of infection to obtain stably transfected cell lines. The effect of stable transfection was verified by RT-qPCR.

### Xenograft model

TE-1 cells in logarithmic growth phase were taken for assays to construct xenograft model. First, eight 4-weeks-old BALB/c-nu mice were separated into two groups at random, namely control group (sh-NC) and circFNDC3B knockdown group (sh-circFNDC3B). The corresponding cells were implanted subcutaneously in the nude mice to form orthotopically transplanted tumors. The mental state and tumor formation of nude mice were observed at any time. Then, the measurement was started when the tumor length exceeded 4 mm, and the measurement was performed every 1 week. At 4 weeks, the mice were sacrificed by de-neck method, transplanted tumors were removed and weighed.

### RT-qPCR

Total RNA was extracted from ESCC tissues or cells by TRIzol method. 1.0 µg total RNA was obtained. cDNA was synthesized by PrimeScript RT enzyme Mix (TaKaRa, Japan). U6 and β-actin were applied as endogenous control genes for miRNA and mRNA, respectively. PCR reaction and data analysis were performed in ABI StepOnePlus /ABI 7500 (Thermo Fisher, USA). The relative levels were computed by 2-^ΔΔCt^. The sequences of primers were as follows:

circFNDC3B: F: 5′-TTCAGACTTGCAAGGTGATTGAAG-3′;

R: 5′-ATACTGTTGTGCAGCTGCTTTT-3′;

liner FNDC3B: F: 5′-ACTGAAAGACCGCCAGATCG-3′;

R: 5′-TCTTGCTCGTCGCTCTGTTT-3′;

miR-370-3p: F: 5′-GCCTGCTGGGGTGGAACCTGGT-3′;

R: 5′-GCAGGGTCCGAGGTATTC-3′;

miR-136-5p: F: 5′-GCCTGGCTGGACAGAGTTG-3′;

R: 5′-GGCTGGGTTGTCATGTGACT-3′;

MYO5A: F: 5′-AGAGAAGTGGGCCTTCTGGT-3′;

R: 5′-GAGCTTCCAAGCCACTTCTG-3′;

β-actin: F: 5′-ATCACTGCCACCCAGAAGAC-3′;

R: 5′-TTTCTAGACGGCAGGTCAGG-3′;

U6: F: 5′-CTCGCTTCGGCAGCACA-3′;

R: 5′-AACGCTTCACGAATTTGCGT-3′.

### Sanger sequencing

The circFNDC3B sequence was obtained using divergent primers sent to Sangon (Shanghai, China) for Sanger sequencing analysis.

### RNase R digestion

We added 1 µl RNase R to 5 µg RNA, then added 1 µl 10X RNase R buffer and 7 µl ddH_2_O. After 15 min in a water bath at 37 °C, linear RNA and circRNA expression was determined by RT-qPCR.

### Actinomycin D assay

For cell counting, 200,000 cells/well were evenly spread in 6-well plates, and allowed to stand for 12 h until the cells adhered. DMEM complete medium containing actinomycin D 2 mg/mL was prepared and the cells were uniformly exchanged. Afterwards, we collected the RNA in the wells at 0, 4, 8, 12, and 24 h, respectively. Finally, we extracted the cellular RNA and stored them at -80 °C for subsequent experiments.

### CCK-8 assay

For CCK-8 assay, 1000 cells were inoculated into each well of 96-well plates. At 0 h, 24 h, 48 h, 72 and 96 h, 10 µL CCK-8 solution (Yeasen, China) was added. The incubation was maintained for 3 h, and the absorbance of each well was determined at 450 nm.

### EdU

According to the instructions of the EdU kit, 50 mM EdU was added to each group of cells after transfection and incubated for 2 h. The cells were later fixed and stained with paraformaldehyde. Nucleic acids in all cells were stained with DAPI. Finally, the fluorescence microscope was used to take photos and images, 5 fields of view were randomly selected for counting.

### Flow cytometry experiment

Cells apoptosis of ESCC was examined via Annexin V-PE/7-AAD kit (Vazyme, China). Briefly, cells were resuspended in 1×binding buffer. Cells were stained with Annexin V-PE and 7-AAD in the darkness. The percentage of apoptotic cells was measured by flow cytometry (San Jose, CA).

### Dual luciferase reporter assay

WT-circFNDC3B, WT-MYO5A, MUT-circFNDC3B and MUT-MYO5A were inserted into the pGL3 vector (GenePharma, China). ESCC cells were inoculated into 24-well plates. Plasmids or 50 nM miR-370-3p/miR-136-5p mimics was cotransfected with ESCC cells applying lipofectamine 3000. Luciferase reporter kit (Promega, USA) was used to measure the Luciferase activity.

### Statistical analysis

Statistical data were analyzed using SPSS 25.0 and shown as the mean ± SD. Student’s t-test and analysis of variance (ANOVA) were used to assess the statistical significance of differences between two or multiple groups. *p* < 0.05 was considered as significant. The receiver operating characteristics (ROC) curves were used to analyze the potential diagnostic value.

## Results

### circFNDC3B is stably and highly expressed in ESCC

**Fig. 1:**
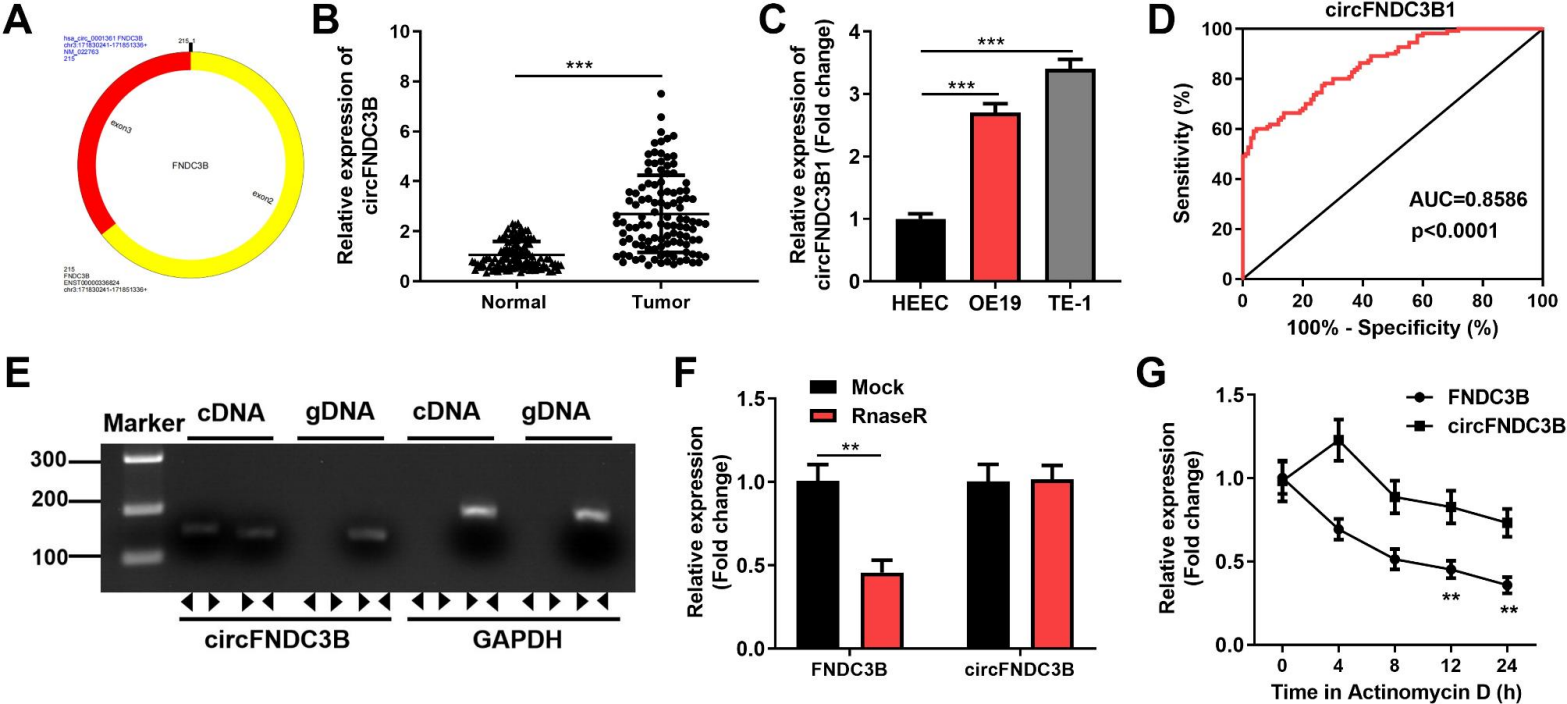
circFNDC3B is stably and highly expressed in ESCC. **(A)**. circFNDC3B schematic diagram of shear. **(B)**. The expression of circFNDC3B in ESCC tissues and paired normal tissues was measured by reverse transcription quantitative polymerase chain reaction (RT-qPCR). **(C)**. The expression of circFNDC3B in ESCC cells (OE19, TE-1) and human normal esophageal epithelial cells HEEC was analyzed by RT-qPCR. **(D)**. The ROC curve of circFNDC3B in ESCC tissue specimens. **(E)**. The presence of circFNDC3B was verified by RT-qPCR and gel electrophoresis. **(F)**. RT-qPCR was used to detect the mRNA expression of circFNDC3B and its linear form before and after digestion by RNase R. **(G)**. The circFNDC3B and its linear form mRNA expression after actinomycin D treatment was analyzed by RT-qPCR. ^**^p < 0.01, ^***^p < 0.001

First, we found that circFNDC3B was made up of exons 2 to 3 of the gene FNDC3B with a spliced maturation sequence length of 215 bp by Sanger sequencing. The circRNA ID was has_circ_0001361, and located at chromosome 3: 171,830,241–171,851,336 (Fig. 1A). Next, the circFNDC3B expression in ESCC was assessed by RT-qPCR. Contrasted to normal tissues, circFNDC3B was significantly raised in ESCC tissues (Fig. 1B). CircFNDC3B was significantly enhanced in OE19 and TE-1 compared to HEEC (Fig. 1C). Then, we discovered the area under the ROC curve (AUC) of circFNDC3B was 0.8586, implying that circFNDC3B had high diagnostic potential (P < 0.0001) (Fig. 1D). Furthermore, we amplified circFNDC3B and FNDC3B from cDNA and gDNA using divergent and convergent primers. The results of agarose gel electrophoresis revealed that circFNDC3B specific bands were magnified in the sample with cDNA as template compared to GAPDH, while no specific bands of circFNDC3B were extended in the sample with gDNA as template, indicating that circFNDC3B was exclusively present in cDNA (Fig. 1E). Subsequently, the RNase R assay disclosed that linear mRNA FNDC3B expression was significantly decreased after RNase R digestion, while circFNDC3B was not, suggesting that circFNDC3B was more resistant (Fig. 1F). The actinomycin D indicated that circFNDC3B was more stable compared to linear mRNA FNDC3B (Fig. 1G). The results illustrated the cyclic character of circFNDC3B. The results also demonstrated that circFNDC3B was significantly heightened in ESCC.

### circFNDC3B promotes cell proliferation and inhibits apoptosis

**Fig. 2:**
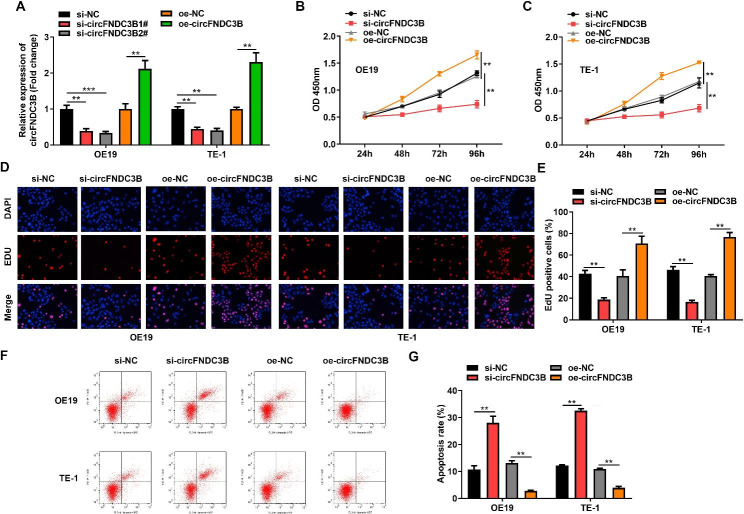
circFNDC3B promotes cell proliferation and inhibits apoptosis. **(A)**. RT-qPCR was used to detect overexpression and knockdown efficiency of circFNDC3B in OE19 and TE-1 cells. **(B-C)**. CCK-8 method was used to assess the absorbance of OE19 and TE-1 cells at 450 nm after overexpression or knockdown of circFNDC3B. **(D-E)**. The proliferation of OE19 and TE-1 cells after circFNDC3B overexpression or knockdown was analyzed by EdU method. **(F-G)**. Flow cytometry was used to detect the apoptosis of OE19 and TE-1 cells after overexpression or knockdown of circFNDC3B. ^**^p < 0.01, ^***^p < 0.001

To explore the biological function of circFNDC3B in ESCC, we used oe-circFNDC3B or si-circFNDC3B (si-circFNDC3B1#, si-circFNDC3B2#) to transfect OE19 and TE-1 cells. The transfection efficiency was verified by RT-qPCR. The analysis revealed that circFNDC3B expression increased significantly after transfection with oe-circFNDC3B in OE19 and TE-1 cells, however, circFNDC3B expression decreased significantly after transfection with si-circFNDC3B in OE19 and TE-1 cells, implying that the transfection was successful (Fig. 2A). Plotting growth curves from CCK-8 experiments revealed that knockdown of circFNDC3B hampered the growth ability of ESCC, whereas circFNDC3B overexpression promoted cell growth (Fig. 2B-C). Similarly, EdU tests suggested that silencing of circFNDC3B reduced the percentage of EdU-positive cells, while introduction of circFNDC3B raised the percentage of EdU-positive cells (Fig. 2D-E). Subsequently, the effect of circFNDC3B on apoptosis was assessed using flow cytometry assay. Inhibition of circFNDC3B significantly enhanced the apoptotic ability of OE19 and TE-1 cells, while insertion of circFNDC3B significantly attenuated the apoptotic ability of OE19 and TE-1 cells (Fig. 2F-G). The above indicated that circFNDC3B advanced ESCC cell proliferation and inhibited ESCC cell apoptosis.

### circFNDC3B targets miR-370-3p/miR-136-5p

**Fig. 3:**
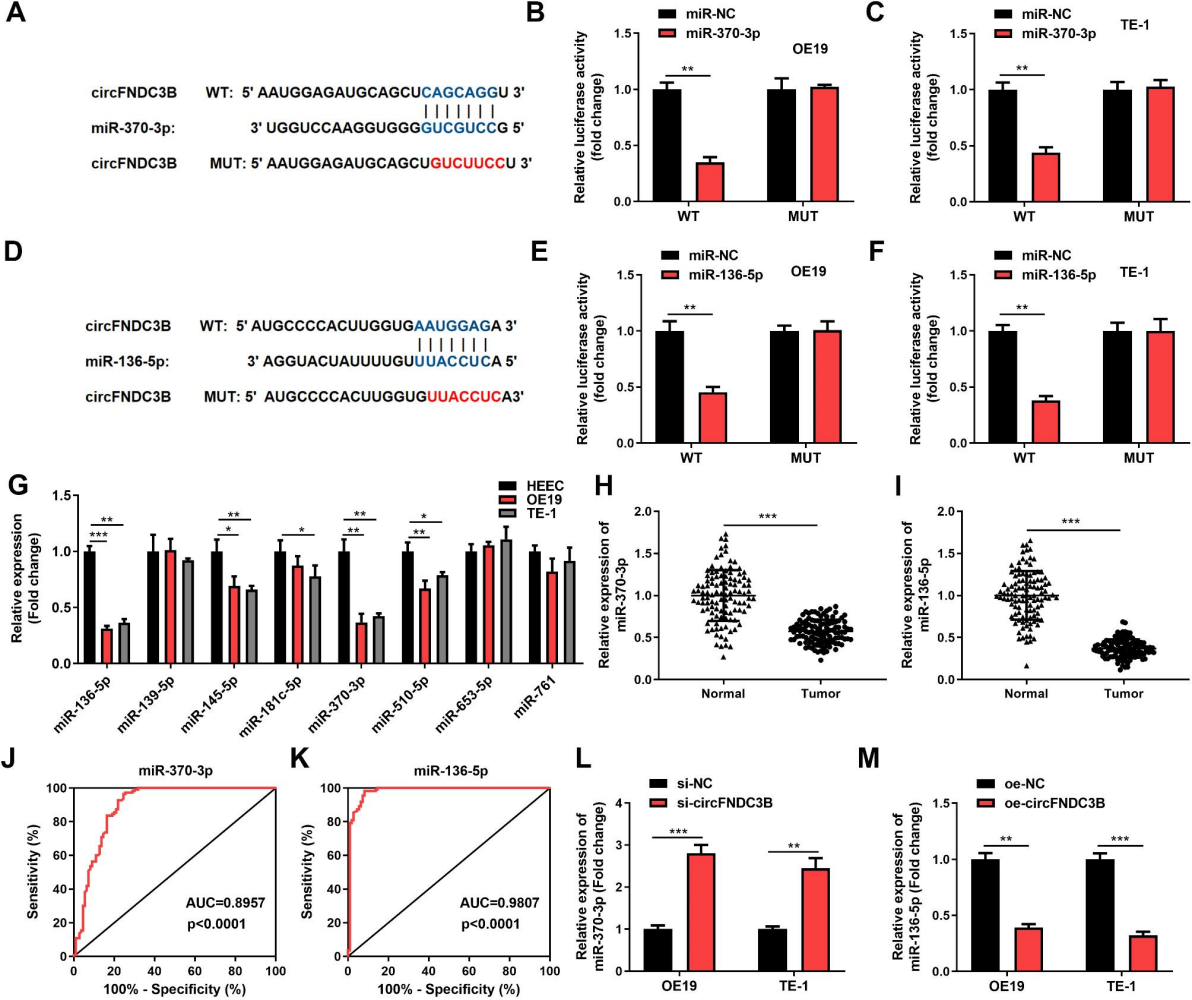
circFNDC3B targets miR-370-3p/miR-136-5p. **(A, D)**. Predicted binding sites of circFNDC3B to miR-370-3p and miR-136-5p by StarBase. **(B-C, E-F)**. Dual luciferase gene reporter assay confirmed the interaction between circFNDC3B and miR-370-3p/miR-136-5p in OE19 and TE-1 cells. **(G)**. Expression of 8 miRNAs with binding sites to circFNDC3B in HEEC, OE19 and TE-1 cells. **(H-I)**. The levels of miR-370-3p and miR-136-5p in ESCC tissues and adjacent normal tissues were detected by RT-qPCR. **(J-K)**. The ROC curve of miR-370-3p and miR-136-5p in ESCC tissue specimens. (L). The expression of miR-370-3p after knockdown of circFNDC3B was measured by RT-qPCR. **(M)**. The expression of miR-136-5p after overexpression of circFNDC3B was analyzed by RT-qPCR. ^*^p < 0.05, ^**^p < 0.01, ^***^p < 0.001

Then, we screened 8 widely studied and novel miRNAs through prediction on StarBase and literature review. Subsequently, we verified the expression of 8 miRNAs in OE19 and TE-1 cells. The expressions of miR-370-3p and miR-136-5p were prominently downregulated in OE19 and TE-1 cells, based on the outcomes of RT-qPCR. Therefore, miR-370-3p and miR-136-5p were selected as research objects (Fig. 3G). Then we found the potential binding sites between circFNDC3B and miR-370-3p/miR-136-5p (Fig. 3A, D). Luciferase assay results disclosed that miR-370-3p/miR-136-5p mimics contributed to an outstanding reduction in WT-circFNDC3B luciferase activity while there was no significantly changed in MUT-circFNDC3B (Fig. 3B, C, E, F). Additionally, miR-370-3p and miR-136-5p were significantly lower in ESCC tissues than in normal tissues (Fig. 3H, I). Low expression of miR-136-5p in ESCC was significantly related to T stage and Lymph node metastasis, while miR-370-3p was significantly linked to T stage (Table 1). The AUC of miR-370-3p and miR-136-5p was 0.8957 and 0.9807, respectively, indicating that they were of great diagnostic value (P < 0.0001) (Fig. 3J, K). Furthermore, miR-370-3p was significantly highly expressed after knockdown of circFNDC3B (Fig. 3L), while miR-136-5p was significantly suppressed by overexpression of circFNDC3B (Fig. 3M). The above suggested an interaction between circFNDC3B and miR-370-3p/miR-136-5p.

**Table 1:** Association of miRNA with clinicopathologic characteristics of esophageal cancer

Clinicopathologic characteristics	miR-370-3p			miR-136-5p		
	Low (n = 73)	High (n = 37)	*p*-value	Low (n = 78)	High (n = 32)	*p*-value
Age (years)			0.7779			0.3378
< 60	26	16		32	10	
≥ 60	47	21		46	22	
Sex			0.2962			0.9164
Male	46	27		52	21	
Female	27	10		26	11	
Pathological typing			0.7400			0.1935
Well/moderate differentiation	26	12		24	14	
Poor differentiation	47	25		54	18	
Gastrointestinal history			0.3011			0.3107
Yes	28	18		35	11	
No	45	19		43	21	
T stage			0.0172*			0.0330*
I–II	47	15		49	13	
III–IV	26	22		29	19	
Lymph node metastasis			0.1197			0.0297*
Yes	43	16		47	12	
No	30	21		31	20	

### Inhibition of circFNDC3B by miR-370-3p/miR-136-5p

**Fig. 4:**
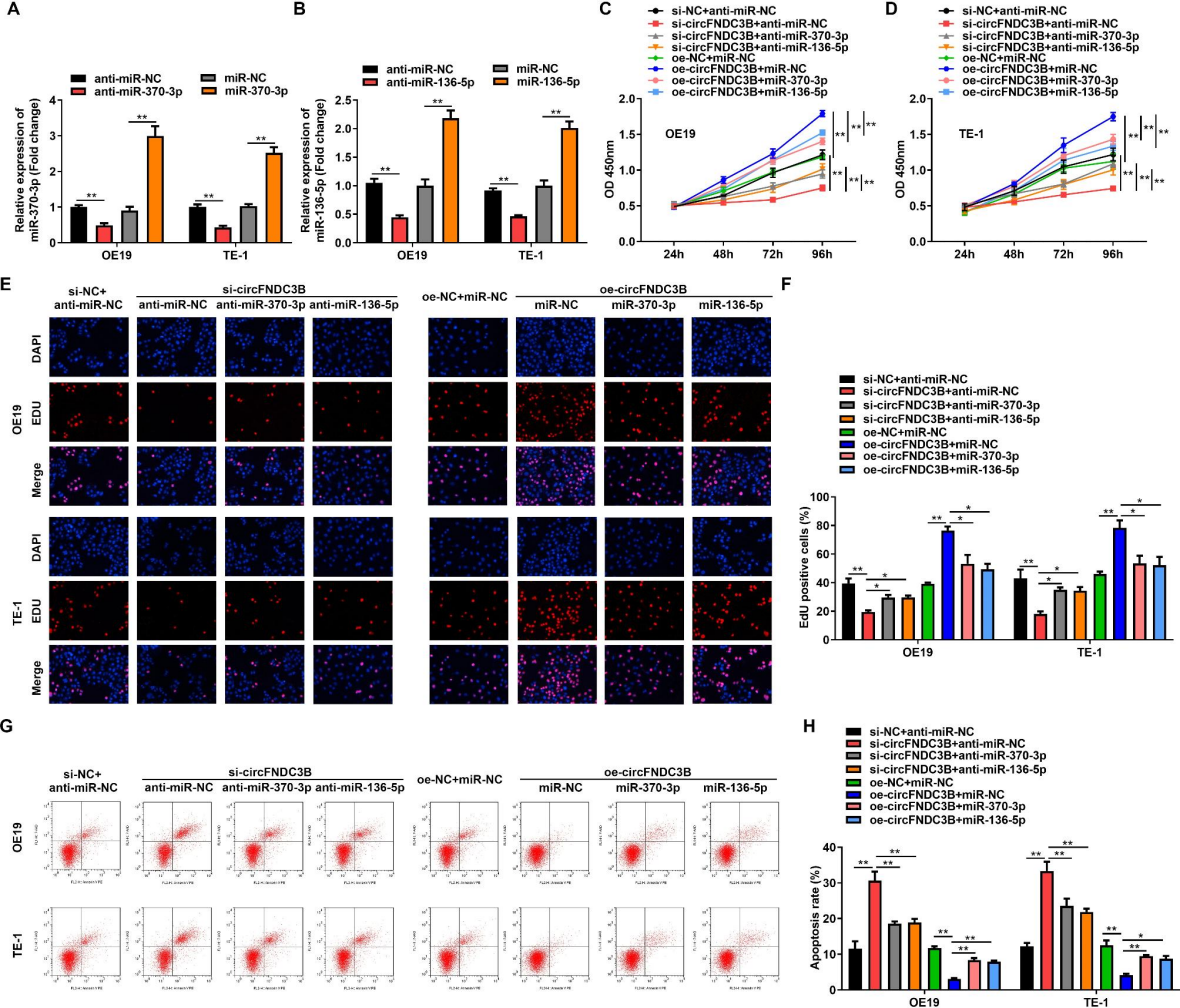
Inhibition of circFNDC3B by miR-370-3p/miR-136-5p. **(A-B)**. RT-qPCR was used to measure the overexpression and knockdown efficiency of miR-370-3p/miR-136-5p in OE19 and TE-1 cells, respectively. **(C-D)**. CCK-8 method was used to assess the absorbance of OE19 and TE-1 cells co-transfected with knockdown circFNDC3B and miR-370-3p or overexpressed circFNDC3B and miR-136-5p at 450 nm. **(E-F)**. The proliferation of OE19 and TE-1 cells co-transfected with knockdown circFNDC3B and miR-370-3p or overexpressed circFNDC3B and miR-136-5p was examined by EdU method. **(G-H)**. Flow cytometry was used to test the apoptosis of OE19 and TE-1 cells co-transfected with silencing circFNDC3B and miR-370-3p or overexpression circFNDC3B and miR-136-5p. ^*^p < 0.05, ^**^p < 0.01

Next, to study the function of miR-370-3p/miR-136-5p in ESCC cells, we transfected OE19 and TE-1 cells with miR-370-3p/miR-136-5p mimics and anti-miR-370-3p/miR-136-5p. The transfection capability was determined employing RT-qPCR. The tests proved that miR-136-5p/miR-370-3p was significantly heightened in OE19 and TE-1 cells after transfection with miR-370-3p/miR-136-5p mimics, however, miR-370-3p/miR-136-5p expression was significantly depressed behind transfection with anti-miR-370-3p/miR-136-5p, indicating that the transfection was perfect (Fig. 4A, B). Subsequently, we examined the biological functions of knockdown or overexpression of miR-370-3p/miR-136-5p in OE19 and TE-1 cells. The findings revealed that anti-miR-370-3p/miR-136-5p rescued the inhibition of si-circFNDC3B on ESCC cell proliferation, while miR-370-3p/miR-136-5p mimic abolished the promotion of oe-circFNDC3B on ESCC cell proliferation (Fig. 4C-F). Later, flow cytometry assays manifested that anti-miR-370-3p/miR-136-5p offset the apoptosis-promoting function of si-circFNDC3B on ESCC cells, while miR-370-3p/miR-136-5p mimic abolished the suppressive role of oe-circFNDC3B on ESCC cell apoptosis (Fig. 4G-H). In combination, circFNDC3B promoted ESCC cell growth and inhibited apoptosis via miR-370-3p/miR-136-5p.

### MYO5A is a common target of mir-370-3p and miR-136-5p

**Fig. 5:**
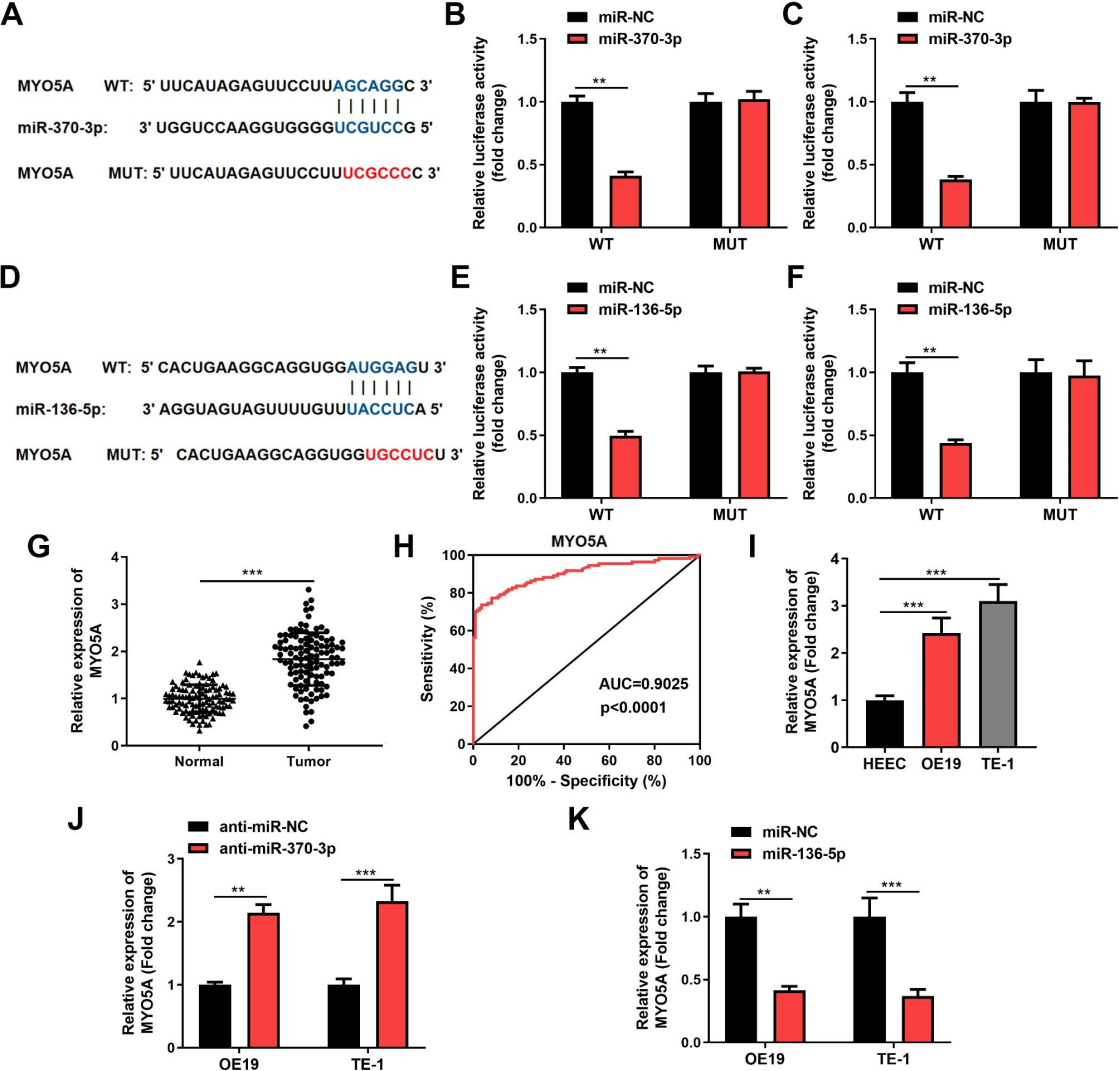
MYO5A is a common target gene of miR-370-3p and miR-136-5p. **(A, D)**. Predicted binding sites of MYO5A to miR-370-3p and miR-136-5p. **(B-C, E-F)**. Dual luciferase gene reporter assay confirmed the interaction between MYO5A and miR-370-3p/miR-136-5p in OE19 and TE-1 cells. **(G)**. The expression of MYO5A in ESCC tissues and adjacent normal tissues were detected by RT-qPCR. **(H)**. The ROC curve of MYO5A in ESCC tissue specimens. **(I)**. The expression of MYO5A in ESCC cells were analyzed by RT-qPCR. (J). The levels of MYO5A were measured by RT-qPCR after knockdown of miR-370-3p. **(K)**. The expression of MYO5A was examined by RT-qPCR after overexpression of miR-136-5p. ^**^p < 0.01, ^***^p < 0.001

To determine the molecular mechanism of miR-370-3p/miR-136-5p in ESCC, we predicted that MYO5A was the target of miR-370-3p/miR-136-5p (Fig. 5A, D). To manifest the prediction, a dual luciferase reporter was constructed. The findings proved that luciferase activity in WT-MYO5A was significantly reduced by miR-370-3p/miR-136-5p insertion, while the activity in MUT-MYO5A was not affected (Fig. 5B, C, E, F). In addition, MYO5A expression in ESCC tissues (Fig. 5G) and cells (Fig. 5I) was significantly higher than that in normal tissues and HEEC. The AUC of MYO5A in ESCC was 0.9025, implying that they were of excellent diagnostic worth (P < 0.0001) (Fig. 5H). Furthermore, MYO5A was significantly highly expressed after knockdown of miR-370-3p (Fig. 5J), while significantly suppressed by introduction of miR-136-5p (Fig. 5K). The above outcomes proved the relationship between MYO5A and miR-370-3p/miR-136-5p.

### Inhibition of MYO5A by miR-370-3p/miR-136-5p

**Fig. 6:**
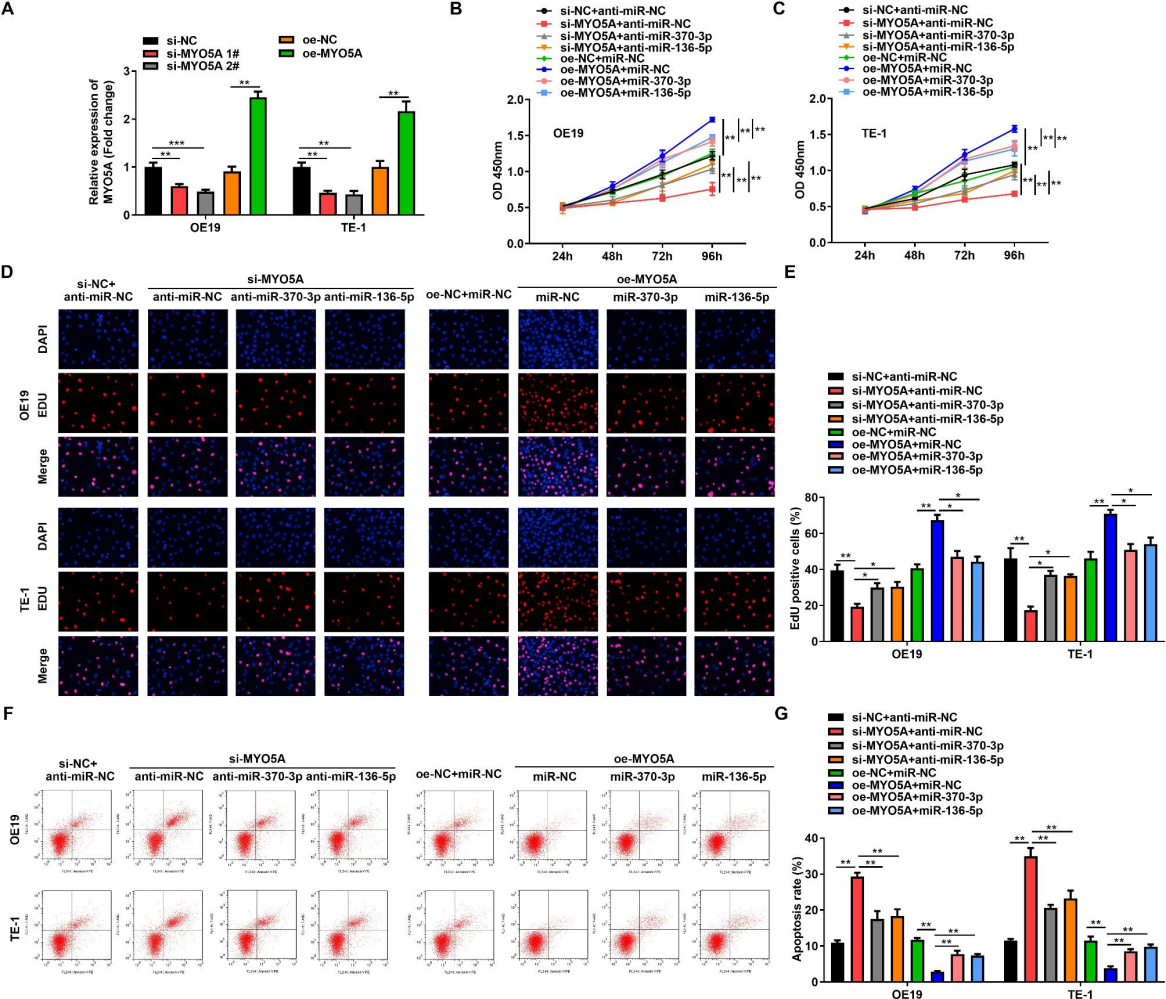
Inhibition of MYO5A by miR-370-3p/miR-136-5p. **(A)**. RT-qPCR was used to assess overexpression and knockdown efficiency of MYO5A in OE19 and TE-1 cells. **(B-C)**. CCK-8 method was applied to test the absorbance of OE19 and TE-1 cells co-transfected with overexpression of MYO5A and miR-370-3p/miR-136-5p or knockdown of MYO5A and miR-370-3p/miR-136-5p at 450 nm. **(D-E)**. The proliferation of OE19 and TE-1 cells co-transfected with overexpression of MYO5A and miR-370-3p/miR-136-5p or knockdown of MYO5A and miR-370-3p/miR-136-5p was analyzed by EdU method. **(F-G)**. Flow cytometry was used to determine the apoptosis of OE19 and TE-1 cells co-transfected with overexpression of MYO5A and miR-370-3p/miR-136-5p or knockdown of MYO5A and miR-370-3p/miR-136-5p. ^*^p < 0.05, ^**^p < 0.01, ^***^p < 0.001

Subsequently, to further verify whether miR-370-3p/miR-136-5p participate in the process of ESCC disease by regulating MYO5A, we transfected OE19 and TE-1 with MYO5A overexpression vector and si-MYO5A (si-MYO5A1#, si-MYO5A2#) and examined the transfection ability using RT-qPCR. The outcomes disclosed that MYO5A expression enhanced in OE19 and TE-1 cells after transfection with oe-MYO5A, and MYO5A expression declined in OE19 and TE-1 cells after transfection with si-MYO5A, suggesting that the transfection was great (Fig. 6A). CCK-8 and EdU consequences revealed that anti-miR-136-5p/miR-370-3p inverted the inhibitory effect of si-MYO5A on ESCC cell growth and miR-370-3p/miR-136-5p mimic abolished the proliferative effect of oe-MYO5A on ESCC cells (Fig. 6B-E). Flow cytometry experiments manifested that anti-miR-370-3p/miR-136-5p offset the promotion of si-MYO5A on ESCC cells apoptosis and miR-370-3p/miR-136-5p mimic rescued the inhibitory of oe-MYO5A on ESCC cell apoptosis (Fig. 6F-G). Taken together, miR-370-3p/miR-136-5p prevented ESCC cell growth and induced apoptosis through MYO5A.

### Knockdown of circFNDC3B inhibits tumor growth in xenograft mice

**Fig. 7:**
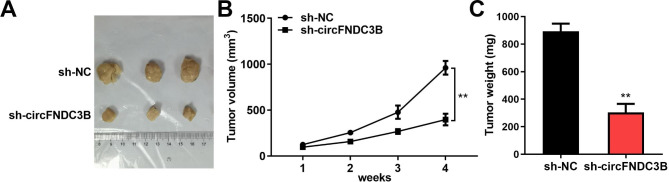
Knockdown of circFNDC3B inhibits tumor growth in xenograft mice. **(A)**. Representative images of xenograft tumors. **(B)**. Changes in tumor volume. **(C)**. Changes in tumor weight. ^**^p < 0.01

Finally, animal xenotransplantation model was established to search the act of circFNDC3B in vivo. circFNDC3B interfering lentiviral vector (sh-circFNDC3B) was constructed and transfected into TE-1 cells. Tumor weight and volume were monitored. It was found that the diameter of the tumor in the negative control group was about 1 cm, while after transfection with sh-circFNDC3B, the diameter of the tumor was near 2.2 cm, as shown in Fig. 7A. In addition, after 4 weeks, the tumor volume of the control group was close to 1000 mm^3^, while the sh-circFNDC3B group was less than 500 mm^3^ (Fig. 7B). Similarly, the tumor weight was approximately 900 mg in the control group and around 300 mg in the sh-circFNDC3B group (Fig. 7C). Therefore, knockdown of circFNDC3B inhibited tumor growth in vivo.

## Discussion

ESCC is a serious malignant tumor with poor prognosis, posing a great threat to the quality of life of humans [1]. ESCC is clinically manifested by insidious onset, rapid development, and easy recurrence and metastasis, resulting in a low 5-year survival rate of about 20% [16]. A major cause of ESCC development is abnormal gene expression in patients [17, 18], however, the exact mechanism of action is unclear. It has been shown that aberrant expression of circRNAs acts a prominent part in the development of many types of cancers, so it is important to explore the function of circRNAs in ESCC to enhance patient survival and improve patient prognosis.

CircRNAs were found sporadically many years ago and believed to be low in abundance as a result of variable splicing errors during transcription [7]. Using high-throughput sequencing and new computational methods, circRNAs from exons or introns were identified as extensive and diverse endogenous eukaryotic non-coding RNAs (ncRNAs) [7], such circular molecules are not sensitive to RNA exonucleases and therefore have a more stable structure than linear RNA [19]. Generally, they were expressed specifically during tissue development and take a vital part in many pathological processes [20, 21], including the transcription of miRNA adsorbed genes regulating RNA-binding proteins and protein translation [22–24]. Due to the structural stability and specificity of circRNAs, more and more research has emphasized the prospective of these ncRNAs as biomarkers for cancer treatment [25]. Recently studies found that circLPAR3 was significantly expressed in ESCC, which was closely connected to clinical stage and lymph node metastasis in ESCC patients, induced ESCC cell metastasis by adjusting the miR-198-MET signaling axis [26]. The study in this paper showed that circFNDC3B was aberrantly highly expressed in ESCC tissues and cells. Zhang et al. in gastric cancer found that large expression of circFNDC3B resulted in higher recurrence rates after treatment in patients with early gastric cancer [27]. Luo et al. disclosed that circFNDC3B was specifically heightened in ESCC tissues [28]. Our study was similar to the outcomes of previous studies. In our paper, we discovered the function of circFNDC3B on ESCC in vitro and in vivo. The findings proved that silencing circFNDC3B in vitro significantly inhibited the growth of ESCC cells and promoted apoptosis. Similarly, knockdown of circFNDC3B inhibited tumor growth in vivo. Therefore, circFNDC3B may be an ESCC-related biomarker.

Then, referring to the competitive endogenous RNA (ceRNA) mechanism verified by numerous scholars, circRNA regulates the target genes expression by interacting with miRNA [29]. We speculated that circFNDC3B participated in the regulation of ESCC by the ceRNA regulation mechanism. Therefore, we predicted and validated that miR-370-3p/miR-136-5p bound to circFNDC3B. miR-370-3p/miR-136-5p has been discussed in numerous tumors. For example, miR-370-3p was significantly diminished in osteosarcoma, inhibited cell proliferation and the EMT process [30]. circ_0020710 enhanced melanoma progression by sponging miR-370-3p [31]. miR-136-5p was lowly expressed in renal cell carcinoma and circTLK1 promoted renal cell carcinoma via sponging miR-136-5p proliferation and metastasis [32]. Reduced expression of miR-136-5p was noticed in gastric cancer, and miR-136-5p offset the effect of hsa_circ_0110389 on the proliferation of gastric cancer cells [33]. Our analysis had similarities with the findings of earlier research. We found that circFNDC3B targeted miR-370-3p/miR-136-5p, which rescued miR-370-3p/miR-136-5p the promotion of circFNDC3B on ESCC cell proliferation and inhibition of circFNDC3B on ESCC cell apoptosis, suggesting that circFNDC3B performed an act in regulating the ESCC cells malignancy by miR-370-3p/miR-136-5p.

Bioinformatics predictions indicated that MYO5A was a downstream target of miR-370-3p/miR-136-5p. MYO5A, also known as Myosin VA, was situated in the q21 region of chromosome 15 and was an actin-dependent motor protein [34]. Initial research of MYO5A concentrated on its role in neurological diseases [35–38]. As research progressed, MYO5A was also found to perform a critical effect in malignant melanoma [39–42]. Subsequently, it was disclosed that MYO5A was associated with metastasis in a variety of cancers. MYO5A was raised in metastatic colorectal cancer and lung cancer tissues and promoted migration of colorectal cancer and lung cancer [43]. Knockdown of LncRNA PART1 led to downregulation of cancer-promoting factor MYO5A and suppression of breast cancer metastasis [44]. Nonetheless, the ability and clinical significance of MYO5A in ESCC are still unclear. In our research, we discovered that MYO5A was highly expressed in ESCC. miR-370-3p / miR-136-5p directly targeted MYO5A. miR-370-3p/miR-136-5p reversed the proliferation-promoting effect of MYO5A on ESCC cells and the inhibitory effect on apoptosis. circFNDC3B targeted MYO5A by sponging miR-370-3p/miR-136-5p thereby promoting the malignant behavior of ESCC cells.

## Conclusion

circFNDC3B regulated the expression of the target gene MYO5A through spongy miR-370-3p/miR-136-5p, thus achieving the cancer-promoting effect on ESCC. This study might provide a therapeutic target for ESCC.

### Electronic supplementary material

Below is the link to the electronic supplementary material.

Supplementary Material 1

## Data Availability

All data used to support the findings of this study are available from the corresponding author upon request.

## Abbreviations

ESCC: Esophageal squamous cell carcinoma
circRNA: Circular RNA
EC: Esophageal cancer
ANOVA: Analysis of variance
ROC: Receiver operating characteristics
ncRNAs: Non-coding RNAs
ceRNA: Competitive endogenous RNA
