# Supplementary figures and images for: circFNDC3B promotes esophageal squamous cell carcinoma progression by targeting MYO5A via miR-370-3p/miR-136-5p
Source: BMC Cancer. 2023 Sep 4;23:821. doi: 10.1186/s12885-023-11314-2 (PMC10476377; doi:10.1186/s12885-023-11314-2)

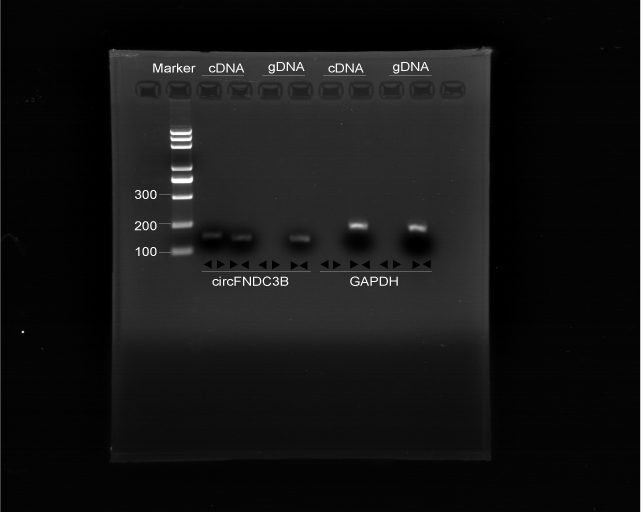

Supplement: Supplementary file 1 — Supplementary Material 1 [file 12885_2023_11314_MOESM1_ESM.tif]
